# Correction to: OA20 A case of rapidly progressive interstitial lung disease in anti-Ro52 positive amyopathic dermatomyositis

**DOI:** 10.1093/rap/rkag068

**Published:** 2026-06-29

This is a correction to: Shiza Chaudhry, Keimee Lopez, Radhikha Raghunath, Olabambo Ogunbambi, OA20 A case of rapidly progressive interstitial lung disease in anti-Ro52 positive amyopathic dermatomyositis, *Rheumatology Advances in Practice*, Volume 9, Issue Supplement_1, November 2025, rkaf111.019, https://doi.org/10.1093/rap/rkaf111.019

In the originally published version of this paper, an erroneous PDF file was inadvertently uploaded.

This has now been corrected.

